# Supplementary material for: Association of a healthy lifestyle with mortality in older people
Source: BMC Geriatr. 2023 Oct 11;23:646. doi: 10.1186/s12877-023-04247-9 (PMC10568769; doi:10.1186/s12877-023-04247-9)
Supplement: Supplementary file 1 — Additional file 1: Supplementary Table 1. Description and prevalence of lifestyle factors in the study population. Table S2. Description of the ascertainment of all-cause and cause specific mortality. Table S3. Baseline characteristics of the study participants by all-cause mortality. Table S4. Hazard ratios of all-cause mortality in relation to individual lifestyle factors (n=11,340). Table S5. Hazard ratios of CVD mortality in relation to individual lifestyle factors (n=11,340). Table S6. Hazard ratios of other mortality in relation to individual lifestyle factors (n=11,340). Table S7. Hazard ratios of cancer mortality in relation to individual lifestyle factors (n=11,340). Table S8. Hazard ratios of all-cause mortality according to combinations of healthy lifestyle factors. Table S9. Hazard ratios of all-cause mortality according to lifestyle categories within demographic, anthropometric and health subgroups. Table S10. Hazard ratios of all-cause mortality according to healthy lifestyle score (excluding former smoker and former drinker). Table S11. Description of an alternative lifestyle score containing multiple levels. Table S12. Hazard ratios of all-cause mortality according to the alternative healthy lifestyle score. Table S13. Hazard ratios of all-cause mortality according to the year-3 healthy lifestyle score. [file 12877_2023_4247_MOESM1_ESM.docx]

**Supplementary Material**

**Table 1 Description and prevalence of lifestyle factors in the study population**

| **Lifestyle factor** | **Points** | **Description** | **Prevalence**  **n (%)** | **Source and definition** |
| --- | --- | --- | --- | --- |
| Smoking | 0  1 | Current smoking: current smoker  Non-smoking: never or former smoker | 290 (2.6)  11050 (97.4) | ASPREE trial baseline medical questionnaire. Participants were asked to report if they were a current, former or never smoker. In the current analyses, participants were classified as either non-smokers or current smokers. Former smokers were categorised together with non-smokers in order to capture current behaviour (regardless of past behaviour). |
| Alcohol consumption | 0  1 | Alcohol consumption: None, low consumption (≤50g/week), high consumption (>100g/week), former drinker  Alcohol consumption: moderate consumption (51-100g/week) | 8815 (77.7)  2525 (22.3) | ASPREE trial baseline medical questionnaire. Participants reported the following detail relating to alcohol consumption: current, former or never; days of drinking per week and average standard drinks per day. Total current alcohol consumption (in grams/week) was calculated by multiplying the days of drinking per week, the average standard drinks per day and the equivalent grams of alcohol in one standard drink. For Australian participants, a standard drink was equivalent to 10 grams. Moderate alcohol consumption was defined as 51-100 grams of alcohol per week, informed by current NHMRC guidelines and findings from a previous study in ASPREE (Neumann et al 2022). |
| Diet | 0  1 | Unhealthy diet: consumption of less than 4 of 7 commonly eaten food groups  Healthy diet: consumption of at least 4 of 7 commonly eaten food groups | 7561 (66.7)  3779 (33.3) | ALSOP year three medical questionnaire, 49-item food frequency questionnaire. Consumption was assessed over five predefined categories of responses ranging from “never/rarely” to “every day or several times a day”. A healthy diet was based on consumption of at least four of seven commonly eaten food groups:  1. Fruits: ≥ 2 times/day  2. Vegetables: ≥ 3 times/day  3. Fish: ≥2 times/week  4. Processed meats: ≤ 1 times/week  5. Unprocessed red meats: ≤ 1.5 times/week  6. Whole grains: ≥ 3 times/day  7. Refined grains: ≤1.5times/day |
| Physical activity | 0  1 | Low physical activity: no or light activity weekly  High physical activity: moderate or vigorous activity weekly | 3749 (33.1)  7591 (66.9) | ALSOP baseline social questionnaire. Participants were asked to report their level of exercise and physical activity in a typical week with options: never/rarely, light, moderate or vigorous physical activity |

**Table 2. Description of the ascertainment of all-cause and cause specific mortality**

| All-cause mortality | Reported deaths were considered to be confirmed upon verification with two independent sources such as family report, clinical record or public death notice. Time-to-event for death was taken as the date of death recorded on the death certificate. |
| --- | --- |
| Cancer mortality  (n=325) | Bladder, blood, brain, breast, cervical, colorectal, kidney, gall bladder, liver, lung, melanoma, Ovary or endometrium, pancreas, prostate, stomach, thyroid, unknown primary, anal, head and neck, mesothelioma, neuroendocrine, other skin, sarcoma |
| Cardiovascular mortality  (n=130) | Cardiac Failure, myocardial infarction, other coronary death, rapid cardiac death, sudden cardiac death, haemorrhagic stroke death, ischemic stroke death, SAH stroke death, stroke death – type unknown |
| Other mortality (n=182) | Dementia, GI disease, liver disease, multi-organ failure/non-specific, other neurological, unknown, renal disease, respiratory (excluding infective pneumonia), sepsis/infection, suicide, trauma, pneumonia |

**Table 3.** **Baseline characteristics of the study participants by all-cause mortality**

| **Characteristics** | **Total ASPREE Cohort**  **(n=19,114)** | **Study cohort**  **(n = 11,340)** | **All-cause mortality**  **(n = 11,340)** | | |
| --- | --- | --- | --- | --- | --- |
|  |  |  | **Yes**  **(n = 702)** | **No**  **(n = 10,638)** | **p-value** |
| Age (years), median (IQR) | 74.0 (71.6-77.7) | 73.9 (71.7-77.3) | 77.8 (73.8-82.1) | 73.7 (71.6-77.0) | <0.001 |
| Male sex, n (%) | 8,332 (43.6) | 5,190 (45.8) | 413 (58.8) | 4,777 (44.1) | <0.001 |
| Living alone, n (%) | 6,252 (33) | 3,468 (30.6) | 264 (37.6) | 3,204 (30.1) | <0.001 |
| <12 years of education, n (%) | 8,636 (45) | 5,380 (47.4) | 356 (50.7) | 5,258 (47.4) | 0.073 |
| IRSAD decile, median (IQR) |  | 6 (4-9) | 6 (4-9) | 6 (4-9) | 0.085 |
| Aspirin allocation, n (%) | 9,525 (50.0) | 5,647 (49.8) | 365 (52.0) | 5,282 (49.7) | 0.229 |
| Medical history, n (%)     Diabetes     Hypertension     Dyslipidaemia     Pre-frailty     Frailty     Depressive symptoms | 2,045 (10.7)  14,195 (74.3)  12,467 (65.2)  7,447 (39.0)  421 (2.2)  1,879 (10) | 1,040 (9.2)  8,392 (74.0)  7,648 (67.4)  3,870 (34.1)  144 (1.3)  972 (8.6) | 95 (13.5)  540 (76.9)  440 (62.7)  347 (49.4)  29 (4.1)  24 (10.04) | 945 (8.9)  7,852 (73.1)  7,208 (67.8)  3,523 (33.1)  115 (1.1)  948 (8.5) | <0.001  0.069  0.005  <0.001  <0.001  0.001 |
| Prescribed medications, n (%)     Statins     Antihypertensives | 6, 470 (34)  10,031 (52.5) | 3,408 (30.1)  5,821 (51.3) | 201 (28.6)  385 (54.8) | 3,207 (30.2)  5,436 (51.1) | 0.397  0.055 |
| Physical Examination     BMI kg/m^2^ mean (SD)     Waist Circumference (cm), mean (SD)     Systolic BP (mm Hg), mean (SD)     Diastolic BP (mm Hg), mean (SD) | 28.1 (4.7)  97.1 (12.9)  139.5 (16.5)  77.3 (9.9) | 27.9 (4.5)  96.8 (12.4)  139.6 (16.2)  77.3 (9.8) | 27.7 (4.8)  98.2 (13.2)  140.5 (16.8)  76.1 (10.2) | 27.9 (4.4)  96.7 (12.3)  139.5 (16.2)  77.3 (9.8) | 0.162  0.002  0.117  0.001 |
| Pathology     HDL (mmol/L), mean (SD)     Non-HDL (mmol/L), mean (SD)     Creatinine (µmol/L), mean (SD)     eGFR (mL/min/1.73m^2^), median (IQR) | 1.6 (0.5)  3.7 (0.9)  80.4 (19.4)  72.9 (62.0-82.7) | 1.6 (0.5)  3.7 (0.9)  79.7 (19.0)  74.3 (64.0-84.2) | 1.6 (0.5)  3.6 (0.9)  84.5 (24.2)  71.8 (60.8-81.8) | 1.6 (0.5)  3.7 (0.9)  79.4 (18.5)  74.5 (64.2-84.4) | 0.941  0.992  <0.001  <0.001 |
| Healthy lifestyle factors^1^, n (%)     No current smoking     High physical activity     Healthy diet     Moderate alcohol consumption | -  -  -  - | 11,050 (97.4)  7,591 (66.9)  3,779 (33.3)  2,525 (22.3) | 666 (94.9)  418 (59.5)  201 (28.6)  125 (17.8) | 10,384 (97.6)  7,173 (67.4)  3,578 (33.6)  2,400 (22.6) | <0.001  <0.001  0.006  0.003 |
| Healthy lifestyle score, n (%)     0     1     2     3     4 | -  -  -  -  - | 72 (0.6)  2,245 (19.8)  5,017 (44.2)  3,358 (29.6)  648 (5.7) | 12 (1.7)  193 (27.5)  299 (42.6)  173 (24.6)  25 (3.6) | 60 (0.6)  2,052 (19.3)  4,718 (44.4)  3,185 (29.9)  623 (5.9) | <0.001 |

Abbreviations: n, sample size; IQR, interquartile range; SD, standard deviation; cm, centimetres; mmol/L, millimoles per litre; µmol/L, micromoles per litre; mL/min, milliliter per minute; eGRF, estimated glomerular filtration rate; IRSAD, Participant Index of Relative Socio-economic Advantage and Disadvantage. Higher score = less disadvantage; BMI, Body Mass Index; BP, blood pressure.

**Table 4. Hazard ratios of all-cause mortality in relation to individual lifestyle factors (n=11,340)**

|  |  |  |  | **Hazard Ratio (95% CI)^1^** | |
| --- | --- | --- | --- | --- | --- |
| **Lifestyle factor** | **n** | **No. Event** | **Incident rate per 1000 py** | **Model 1** | **Model 2** |
| **Smoking** |  |  |  |  |  |
| 0 | 290 | 36 | 18.90 | 1 [Reference] | 1 [Reference] |
| 1 | 11050 | 666 | 8.99 | 0.40 (0.28, 0.55) | 0.42 (0.30, 0.59) |
| **Alcohol consumption** |  |  |  |  |  |
| 0 | 8815 | 145 | 2.45 | 1 [Reference] | 1 [Reference] |
| 1 | 2525 | 30 | 1.77 | 0.82 (0.55, 1.21) | 0.92 (0.62, 1.37) |
| **Diet** |  |  |  |  |  |
| 0 | 7561 | 501 | 9.91 | 1 [Reference] | 1 [Reference] |
| 1 | 3779 | 201 | 7.90 | 0.86 (0.73, 1.01) | 0.90 (0.76, 1.06) |
| **Physical activity** |  |  |  |  |  |
| 0 | 3749 | 284 | 11.29 | 1 [Reference] | 1 [Reference] |
| 1 | 7591 | 418 | 8.22 | 0.81 (0.70, 0.95) | 0.84 (0.72, 0.98) |

^1^ Model 1 adjusted for age, sex and aspirin treatment allocation; Model 2 adjusted for Model 1 plus mutually adjusted for other lifestyle factors, education, living status, socioeconomic status

**Table 5. Hazard ratios of CVD mortality in relation to individual lifestyle factors (n=11,340)**

|  |  |  |  | **Hazard Ratio (95% CI)^1^** | |
| --- | --- | --- | --- | --- | --- |
| **Lifestyle factor** | **n** | **No. Event** | **Incident rate per 1000 py** | **Model 1** | **Model 2** |
| **Smoking** |  |  |  |  |  |
| 0 | 290 | 11 | 5.78 | 1 [Reference] | 1 [Reference] |
| 1 | 11050 | 164 | 2.21 | 0.30 (0.16, 0.56) | 0.33 (0.18, 0.62) |
| **Alcohol consumption** |  |  |  |  |  |
| 0 | 8815 | 145 | 2.45 | 1 [Reference] | 1 [Reference] |
| 1 | 2525 | 30 | 1.77 | 0.82 (0.55, 1.21) | 0.92 (0.62, 1.37) |
| **Diet** |  |  |  |  |  |
| 0 | 7561 | 127 | 2.51 | 1 [Reference] | 1 [Reference] |
| 1 | 3779 | 48 | 1.89 | 0.80 (0.57, 1.12) | 0.87 (0.62, 1.22) |
| **Physical activity** |  |  |  |  |  |
| 0 | 3749 | 82 | 3.26 | 1 [Reference] | 1 [Reference] |
| 1 | 7591 | 93 | 1.83 | 0.66 (0.49, 0.90) | 0.69 (0.51, 0.94) |

^1^ Model 1 adjusted for age, sex and aspirin treatment allocation; Model 2 adjusted for Model 1 plus mutually adjusted for other lifestyle factors, education, living status, socioeconomic status

**Table 6. Hazard ratios of other mortality in relation to individual lifestyle factors (n=11,340)**

|  |  |  |  | **Hazard Ratio (95% CI)^1^** | |
| --- | --- | --- | --- | --- | --- |
| **Lifestyle factor** | **n** | **No. Event** | **Incident rate per 1000 py** | **Model 1** | **Model 2** |
| **Smoking** |  |  |  |  |  |
| 0 | 290 | 7 | 3.68 | 1 [Reference] | 1 [Reference] |
| 1 | 11050 | 182 | 2.46 | 0.52 (0.25, 1.12) | 0.57 (0.26, 1.21) |
| **Alcohol consumption** |  |  |  |  |  |
| 0 | 8815 | 163 | 2.76 | 1 [Reference] | 1 [Reference] |
| 1 | 2525 | 26 | 1.54 | 0.61 (0.41, 0.93) | 0.64 (0.42, 0.97) |
| **Diet** |  |  |  |  |  |
| 0 | 7561 | 136 | 2.69 | 1 [Reference] | 1 [Reference] |
| 1 | 3779 | 53 | 2.08 | 0.86 (0.62, 1.18) | 0.89 (0.65, 1.23) |
| **Physical activity** |  |  |  |  |  |
| 0 | 3749 | 80 | 3.18 | 1 [Reference] | 1 [Reference] |
| 1 | 7591 | 109 | 2.14 | 0.77 (0.58, 1.04) | 0.80 (0.59, 1.07) |

^1^ Model 1 adjusted for age, sex and aspirin treatment allocation; Model 2 adjusted for Model 1 plus mutually adjusted for other lifestyle factors, education, living status, socioeconomic status

**Table 7. Hazard ratios of cancer mortality in relation to individual lifestyle factors (n=11,340)**

|  |  |  |  | **Hazard Ratio (95% CI)^1^** | |
| --- | --- | --- | --- | --- | --- |
| **Lifestyle factor** | **n** | **No. Event** | **Incident rate per 1000 py** | **Model 1** | **Model 2** |
| **Smoking** |  |  |  |  |  |
| 0 | 290 | 18 | 9.45 | 1 [Reference] | 1 [Reference] |
| 1 | 11050 | 319 | 4.30 | 0.40 (0.25, 0.65) | 0.41 (0.26, 0.67) |
| **Alcohol consumption** |  |  |  |  |  |
| 0 | 8815 | 268 | 4.53 | 1 [Reference] | 1 [Reference] |
| 1 | 2525 | 690 | 4.08 | 0.94 (0.72, 1.23) | 0.98 (0.75, 1.28) |
| **Diet** |  |  |  |  |  |
| 0 | 7561 | 237 | 4.69 | 1 [Reference] | 1 [Reference] |
| 1 | 3779 | 100 | 3.93 | 0.90 (0.71, 1.14) | 0.92 (0.72, 1.16) |
| **Physical activity** |  |  |  |  |  |
| 0 | 3749 | 122 | 4.85 | 1 [Reference] | 1 [Reference] |
| 1 | 7591 | 215 | 4.22 | 0.93 (0.74, 1.17) | 0.95 (0.76, 1.20) |

^1^ Model 1 adjusted for age, sex and aspirin treatment allocation; Model 2 adjusted for Model 1 plus mutually adjusted for other lifestyle factors, education, living status, socioeconomic status

**Table 8. Hazard ratios of all-cause mortality according to combinations of healthy lifestyle factors**

| **Combination of factors** | **N (%)**  **n=11,340** | **No. Event** | **Incident rate per 1000 py** | **Hazard Ratios (95% CI)** | |
| --- | --- | --- | --- | --- | --- |
|  |  |  |  | **Model 1** | **Model 2** |
|  |  |  |  |  |  |
| **0 or 1 factors** | 2317 (20.4) | 205 | 11.53 | 1 [Reference] | 1 [Reference] |
| **2 factors** |  |  |  |  |  |
| Non-smoking + moderate alcohol | 430 (3.8) | 22 | 7.59 | 0.60 (0.39, 0.93) | 0.61 (0.39, 0.95) |
| Non-smoking + healthy diet | 861 (7.6) | 57 | 9.82 | 0.78 (0.58, 1.04) | 0.79 (0.59, 1.06) |
| Non-smoking + high physical activity | 3654 (32.2) | 212 | 8.67 | 0.71 (0.59, 0.87) | 0.72 (0.59, 0.87) |
| Moderate alcohol + healthy diet | 7 (0.1) | 1 | - | - | - |
| Moderate alcohol + high physical activity | 17 (0.2) | 3 | - | - | - |
| Healthy diet + high physical activity | 48 (0.4) | 4 | - | - | - |
| **3 factors** |  |  |  |  |  |
| Non-smoking + moderate alcohol + healthy diet | 240 (2.1) | 12 | 7.43 | 0.62 (0.35, 1.13) | 0.65 (0.36, 1.16) |
| Non-smoking + moderate alcohol + high physical activity | 1159 (10.2) | 61 | 7.91 | 0.70 (0.53, 0.86) | 0.73 (0.54, 0.88) |
| Non-smoking + healthy diet + high physical activity | 1952 (17.2) | 100 | 7.62 | 0.68 (0.53, 0.86) | 0.69 (0.54, 0.88) |
| Moderate alcohol + healthy diet + high physical activity | 7 (0.1) | 0 | - | - | - |
| **4 factors** |  |  |  |  |  |
| Non-smoking + moderate alcohol + healthy diet + high physical activity | 648 (5.7) | 25 | 5.69 | 0.53 (0.35, 0.81) | 0.57 (0.37, 0.86) |

^1^ Model 1 adjusted for age, sex and aspirin treatment allocation; Model 2 adjusted for Model 1 plus education, living status, socioeconomic status

*****HRs calculated for combinations >2% with a minimum of 10 events**.** Within the reference group of individuals with 0 or 1 healthy lifestyle factors, 0.63% had 0 factors, 0.93% had only high physical activity, 18.6% had only non-smoking, 0.15% had only moderate alcohol and 0.14% had only healthy diet.

**Table 9. Hazard ratios of all-cause mortality according to lifestyle categories within demographic, anthropometric and health subgroups**

|  | **N (%)** | **No. Event** | **Incident rate per 1000 py** | **Hazard ratio (95% CI)^1^** | **P-value for overall interaction** |
| --- | --- | --- | --- | --- | --- |
| **Sex** |  |  |  |  | 0.20 |
| *Male* |  |  |  |  |  |
| Unfavourable | 938 (18.1) | 120 | 19.26 | 1 [Reference] |  |
| Moderate | 2,428 (46.8) | 172 | 10.63 | 0.65 (0.51, 0.82) |  |
| Favourable | 1,824 (35.1) | 121 | 9.94 | 0.65 (0.50, 0.84) |  |
| *Female* |  |  |  |  |  |
| Unfavourable | 1,379 (22.4) | 85 | 9.17 | 1 [Reference] |  |
| Moderate | 2,589 (42.1) | 127 | 7.28 | 0.87 (0.66, 1.15) |  |
| Favourable | 2,182 (35.5) | 77 | 5.24 | 0.72 (0.52, 0.98) |  |
| **Median Age** |  |  |  |  |  |
| *70-73* |  |  |  |  | 0.30 |
| Unfavourable | 1,052 (18.2) | 53 | 7.57 | 1 [Reference] |  |
| Moderate | 2,517 (43.5) | 77 | 3.66 | 0.59 (0.42, 0.84) |  |
| Favourable | 2,222 (38.4) | 62 | 3.26 | 0.56 (0.39, 0.82) |  |
| *74+* |  |  |  |  |  |
| Unfavourable | 1,265 (22.8) | 152 | 17.89 | 1 [Reference] |  |
| Moderate | 2,500 (45.1) | 222 | 13.21 | 0.71 (0.58, 0.88) |  |
| Favourable | 1,784 (32.1) | 136 | 11.30 | 0.62 (0.49, 0.78) |  |
| **Education** |  |  |  |  | 0.93 |
| *≥12* |  |  |  |  |  |
| Unfavourable | 1,092 (18.3) | 90 | 12.19 | 1 [Reference] |  |
| Moderate | 2,507 (42.1) | 144 | 8.56 | 0.75 (0.58, 0.98) |  |
| Favourable | 2,361 (39.6) | 112 | 7.08 | 0.69 (0.52, 0.91) |  |
| *<12* |  |  |  |  |  |
| Unfavourable | 1,225 (22.8) | 115 | 11.81 | 1 [Reference] |  |
| Moderate | 2,510 (46.6) | 155 | 7.88 | 0.72 (0.57, 0.92) |  |
| Favourable | 1,645 (30.6) | 86 | 6.29 | 0.67 (0.51, 0.89) |  |
| **BMI** |  |  |  |  | 0.36 |
| *<25* |  |  |  |  |  |
| Unfavourable | 481 (16.0) | 53 | 16.47 | 1 [Reference] |  |
| Moderate | 1,286 (42.9) | 87 | 10.05 | 0.78 (0.55, 1.10) |  |
| Favourable | 1,233 (41.1) | 59 | 7.00 | 0.58 (0.40, 0.85) |  |
| *≥25* |  |  |  |  |  |
| Unfavourable | 1,823 (22.0) | 152 | 12.47 | 1 [Reference] |  |
| Moderate | 3,707 (44.7) | 210 | 8.46 | 0.71 (0.58, 0.88) |  |
| Favourable | 2,758 (33.3) | 137 | 7.48 | 0.71 (0.56, 0.90) |  |
| **Aspirin treatment allocation** |  |  |  |  | 0.07 |
| *Treatment* |  |  |  |  |  |
| Unfavourable | 1,131 (20.0) | 120 | 15.84 | 1 [Reference] |  |
| Moderate | 2,519 (44.6) | 153 | 9.08 | 0.64 (0.51, 0.82) |  |
| Favourable | 1,997 (35.4) | 92 | 6.87 | 0.53 (0.41, 0.70) |  |
| *Placebo* |  |  |  |  |  |
| Unfavourable | 1,186 (20.8) | 85 | 10.73 | 1 [Reference] |  |
| Moderate | 2,498 (43.9) | 146 | 8.71 | 0.86 (0.66, 1.12) |  |
| Favourable | 2,009 (35.3) | 106 | 7.85 | 0.89 (0.66, 1.18) |  |
| **Diabetes Miletus** |  |  |  |  |  |
| *Yes* |  |  |  |  |  |
| Unfavourable | 292 (28.1) | 35 | 18.53 | 1 [Reference] | 0.23 |
| Moderate | 459 (44.1) | 31 | 10.44 | 0.64 (0.39, 1.04) |  |
| Favourable | 289 (27.8) | 29 | 15.37 | 0.96 (0.58, 1.57) |  |
| *No* |  |  |  |  |  |
| Unfavourable | 2,025 (19.7) | 170 | 12.49 | 1 [Reference] |  |
| Moderate | 4,558 (44.2) | 268 | 8.74 | 0.76 (0.63, 0.92) |  |
| Favourable | 3,717 (36.1) | 169 | 6.76 | 0.67 (0.54, 0.83) |  |
| **Hypertension** |  |  |  |  | 0.65 |
| *Yes* |  |  |  |  |  |
| Unfavourable | 1,864 (22.3) | 171 | 13.67 | 1 [Reference] |  |
| Moderate | 3,723 (44.4) | 229 | 9.17 | 0.73 (0.60, 0.90) |  |
| Favourable | 2,802 (33.4) | 140 | 7.42 | 0.65 (0.52, 0.82) |  |
| *No* |  |  |  |  |  |
| Unfavourable | 450 (15.3) | 34 | 11.38 | 1 [Reference] |  |
| Moderate | 1,294 (43.9) | 70 | 8.09 | 0.74 (0.49, 1.13) |  |
| Favourable | 1,204 (40.8) | 58 | 7.24 | 0.76 (0.49, 1.17) |  |

^1^HRs adjusted for age, sex, aspirin treatment allocation, education, living status, socioeconomic status

**Sensitivity analyses**

**Table 10. Hazard ratios of all-cause mortality according to healthy lifestyle score (excluding former smoker and former drinker)**

|  |  |  |  | **Hazard Ratio (95% CI)^1^** | |
| --- | --- | --- | --- | --- | --- |
|  | **N 10,258** | **No. Event** | **Incident rate per 1000 py** | **Model 1** | **Model 2** |
| **Healthy lifestyle score categories** |  |  |  |  |  |
| Unfavourable (≤1) | 2,045 | 173 | 12.64 | 1 [Reference] | 1 [Reference] |
| Moderate (2) | 4,531 | 257 | 8.44 | 0.72 (0.59, 0.87) | 0.72 (0.60, 0.88) |
| Favourable (≥3) | 3,682 | 180 | 7.29 | 0.69 (0.56, 0.85) | 0.71 (0.58, 0.88) |
| p-value for trend |  |  |  | 0.001 | 0.001 |

^1^Model 1 adjusted for age, sex and aspirin treatment allocation; Model 2 adjusted for model 1 plus living status, education, socioeconomic status

**Table 11. Description of an alternative lifestyle score containing multiple levels**

| **Variable** | **Score** |
| --- | --- |
| Smoking | 1 = Never  .67 = Former quit >=15 yrs  .33 = Former quit <15 yrs  0 = Current |
| Alcohol | 1 = Never  .75 = Current Adherence  .5 = Former adherence  .25 = Former non-adherence  0 = Current non-adherence |
| Diet (fruit and vegetable consumption only)  *Quartiles of serves per week categorised into top and bottom 25%, middle 50% - top 25% equates to approx. recommendation of >5 serves per day* | 1 = >33 (> 4.7 serves per day)  0.5 = 21 – 33p/w (3-4.7 serves per day)  0 = <21pw (<3 serves per day) |
| Physical activity | 1 = Vigorous weekly PA  .5 = Moderate weekly PA  0 = Low/none weekly PA |
|  |  |
| **Lifestyle Score range** | Average sum of lifestyle factor scores range: 0-1 |
| **Categorical Lifestyle Score** | Low: 1^st^ quintile  Moderate: 2^nd^ to 4^th^ quintile  Favourable: 5^th^ quintile |

**Table 12. Hazard ratios of all-cause mortality according to the alternative healthy lifestyle score**

|  |  |  |  | **Hazard Ratio (95% CI)^1^** | |
| --- | --- | --- | --- | --- | --- |
|  | **N 11,340** | **No. Event** | **Incident rate per 1000 py** | **Model 1** | **Model 2** |
| **Healthy lifestyle score categories** |  |  |  |  |  |
| Unfavourable (Q1) | 2,317 | 170 | 11.76 | 1 [Reference] | 1 [Reference] |
| Moderate (Q2-4) | 5,017 | 350 | 8.40 | 0.79 (0.66, 0.96) | 0.80 (0.66, 0.96) |
| Favourable (Q5) | 4,006 | 92 | 6.69 | 0.65 (0.50, 0.84) | 0.66 (0.51, 0.85) |
| p-value for trend |  |  |  | 0.001 | 0.001 |

^1^Model 1 adjusted for age, sex and aspirin treatment allocation; Model 2 adjusted for model 1 plus living status, education, socioeconomic status

**Table 13. Hazard ratios of all-cause mortality according to the year-3 healthy lifestyle score**

|  |  |  |  | **Hazard Ratio (95% CI)^1^** | |
| --- | --- | --- | --- | --- | --- |
|  | **N 11,340** | **No. Event** | **Incident rate per 1000 py** | **Model 1** | **Model 2** |
| **Healthy lifestyle score categories** |  |  |  |  |  |
| Unfavourable | 2,240 | 200 | 13.28 | 1 [Reference] | 1 [Reference] |
| Moderate | 4,383 | 234 | 7.87 | 0.67 (0.55, 0.81) | 0.67 (0.55, 0.81) |
| Favourable | 2,830 | 124 | 6.39 | 0.60 (0.48, 0.76) | 0.62 (0.49, 0.78) |
| p-value for trend |  |  |  | 0.001 | 0.001 |

^1^Model 1 adjusted for age, sex and aspirin treatment allocation; Model 2 adjusted for model 1 plus living status, education, socioeconomic status

**Alternative alcohol variable**

The alternative alcohol categorisation of low/moderate/none vs high was inversely associated all-cause mortality (HR=1.01 [95% CI 0.85, 1.20]), although not statistically significant. Nonetheless, when combined as part of the lifestyle score, full covariate adjusted HRs for the risk of all-cause mortality among those with moderate (HR=0.68 [95% CI 0.54, 0.89]) and favourable (HR=0.64 [0.51, 0.82]) lifestyle remained equivocal.
